# A link between energy metabolism and plant host adaptation states in the two-spotted spider mite, *Tetranychus urticae* (Koch)

**DOI:** 10.1038/s41598-023-46589-9

**Published:** 2023-11-07

**Authors:** Jorden Maglov, Min Yi Feng, Dorothy Lin, Kennedy Barkhouse, Anton Alexander, Miodrag Grbic, Vladimir Zhurov, Vojislava Grbic, Slavica Tudzarova

**Affiliations:** 1 Department of Biology, The University of Western Ontario, London, N6A 5B7 Canada; 2 Larry L. Hillblom Islet Research Center, University of California, Los Angeles, CA 90095 USA

**Keywords:** Metabolism, Evolution, Molecular biology

## Abstract

Energy metabolism is a highly conserved process that balances generation of cellular energy and maintenance of redox homeostasis. It consists of five interconnected pathways: glycolysis, tricarboxylic acid cycle, pentose phosphate, trans-sulfuration, and NAD+ biosynthesis pathways. Environmental stress rewires cellular energy metabolism. Type-2 diabetes is a well-studied energy metabolism rewiring state in human pancreatic β-cells where glucose metabolism is uncoupled from insulin secretion. The two-spotted spider mite, *Tetranychus urticae* (Koch)*,* exhibits a remarkable ability to adapt to environmental stress. Upon transfer to unfavourable plant hosts, mites experience extreme xenobiotic stress that dramatically affects their survivorship and fecundity. However, within 25 generations, mites adapt to the xenobiotic stress and restore their fitness. Mites’ ability to withstand long-term xenobiotic stress raises a question of their energy metabolism states during host adaptation. Here, we compared the transcriptional responses of five energy metabolism pathways between host-adapted and non-adapted mites while using responses in human pancreatic islet donors to model these pathways under stress. We found that non-adapted mites and human pancreatic β-cells responded in a similar manner to host plant transfer and diabetogenic stress respectively, where redox homeostasis maintenance was favoured over energy generation. Remarkably, we found that upon host-adaptation, mite energy metabolic states were restored to normal. These findings suggest that genes involved in energy metabolism can serve as molecular markers for mite host-adaptation.

## Introduction

**Figure 1:**
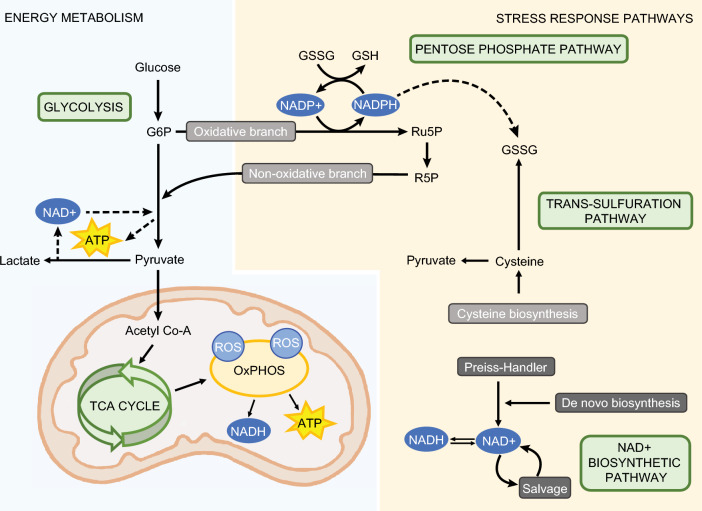
Energy metabolism and stress response pathways in a healthy cell. Pyruvate generated from glucose through glycolysis is used as a substrate for the mitochondrial Tricarboxylic acid (TCA) cycle that is coupled with Oxidative phosphorylation (OxPHOS) to generate ATP, ROS and NADH. NAD is mainly generated by the Salvage pathway. Glycolysis intermediates participate in the non-oxidative pentose phosphate pathway (PPP) to reduce oxidized glutathione (GSS) synthesized by the trans-sulfuration pathway. The oxidative branch of the PPP supports biosynthesis of nucleotide precursors of nucleic acids.

Simple monosaccharides such as glucose are evolutionarily conserved energy sources. In glycolysis, the energy within the sugar molecule is converted to adenosine triphosphate (ATP) to fuel cellular processes. The conversion of glucose to ATP occurs across different cellular compartments and is divided into several pathways, Fig. 1. Glucose undergoes initial metabolism through the glycolysis pathway in the cytoplasm to produce pyruvate molecules that are then converted to acetyl coenzyme A (acetyl CoA) in the mitochondria to initiate the tricarboxylic acid (TCA) cycle. The TCA cycle utilizes a stepwise process to capture the energy stored within the chemical bonds of acetyl CoA in the form of high-energy intermediate molecules. The trapped energy from the TCA cycle is harnessed by oxidative phosphorylation (OxPHOS) through electron transport from the TCA cycle energy precursors. The energy is transformed into a highly usable form of cellular energy, resulting in the production of 32-36 molecules of ATP from a single glucose molecule. Besides ATP molecules, reduced nicotinamide adenine dinucleotide (NADH) and reactive oxygen species (ROS) form as byproducts of OxPHOS^1,2^. Although ROS can serve as signaling molecules at lower concentrations^3,4^, its accumulation within cells can lead to oxidative stress and trigger DNA damage and cell death. As such, excess ROS is detoxified through the pentose phosphate pathway (PPP) that runs in parallel to glycolysis. The PPP utilizes glucose 6-phosphate (G6P) from the glycolysis pathway and shunts carbons back to it. It forms ribose 5-phosphate (R5P), a precursor for DNA repair and DNA synthesis, and enables nicotinamide phosphate (NADPH)-mediated detoxification of ROS by the antioxidant glutathione (GSH)^5^. GSH is primarily synthesized by the trans-sulfuration (TS) pathway from the amino acid cysteine^6^. Besides ROS, OxPHOS generates NADH as well^1,7^. NAD^+^ is mainly generated via glycolysis and NADH via the TCA cycle, so the ratio between NAD^+^/NADH indicates the contribution of glycolysis versus TCA cycle activity. NAD can be synthesized by several pathways including the *de novo-*, Preiss-Handler- and salvage pathways^8^. Thus, metabolic homeostasis depends on tight coordination between five pathways that enables the cell to utilize chemical energy and balance out by-products that form along the way.

Various endogenous and exogenous (environmental) stressors lead to energy rewiring to either respond to increased anabolic demand (growth and replication) or provide adaptation to stress at the expense of dissipation of chemical energy or lower ATP yield. Xenobiotic stress is one of the factors that modifies cellular homeostasis. Xenobiotics are a diverse family of pollutants with a global dispersion and consistent bioaccumulation^9,10^. Cells exposed to xenobiotics undergo an oxidative stress^11,12^. While all cells have to balance their energy metabolism and redox status, human pancreatic β-cells that have poor antioxidant defense machinery^13–15^, rely on the remodeling of the metabolism in order to survive stress conditions^11,16,17^. Furthermore, energy metabolism and its rewiring under stress is exceptionally well described in β-cells as a misbalance in energy pathways leads to a pathological cellular phenotype, type-2 diabetes (T2D), characterized by the decoupling of glucose metabolism from insulin secretion. Under T2D stress, glucose metabolism in β-cells is redirected from the TCA cycle to the production of lactate, Fig. 1. As a consequence, there is a drop in mitochondrial ATP production. A reduced ATP/ADP ratio will fail to depolarize the cell membrane and induce Ca2+ influx necessary for insulin secretion^18^. Increased conversion of pyruvate to lactate leads to increased NAD^+^ and an increased NAD^+^/NADH ratio that perturbs the cellular redox balance. Furthermore, the backflow of PPP metabolites to glycolysis becomes restricted, centering the stress response to regeneration of GSH and antioxidant defenses^19,20^.

Recent data implicated xenobiotics as a contributing factor driving impaired glucose homeostasis, β-cell dysfunction, and altered metabolic and oxidative stress pathways in pancreatic islets^21^. While human exposure to xenobiotics leads to a failed adaptation and morbidity as in T2D, there are organisms, like the two spotted spider mite (TSSM) *Tetranychus urticae* that can readily adapt to xenobiotic stress^22,23^. TSSM is a polyphagous arthropod pest with more than 1100 documented plant hosts^24^. Such a wide host range implies that mites can disarm a broad range of plant allelochemicals. However, individual mite populations can only feed on a select number of plant hosts but have the ability to adapt to new plant hosts within 5–25 generations^25,26^. TSSM’s ability to rapidly adapt to xenobiotic challenge upon host transfer raises a question of mite energy metabolic states when they are exposed to xenobiotic stress. Availability of the fully sequenced and annotated genome of *T. urticae*^27^ and datasets that document mite transcriptional responses to xenobiotic challenge enabled a comparative study of energy metabolic responses to xenobiotics. We aimed at elucidating the evolutionary conserved metabolic adaptation to xenobiotic stress that is shared between human β-cells and mites. We performed a comprehensive analysis of transcriptional changes of energy metabolic pathways in xenobiotically adapted and non-adapted TSSM populations and have compared them to transcriptional changes of living human islets from donors with impaired glucose tolerance (IGT) and sincere T2D^28^. Our results indicate a significant overlap in energy metabolism between human T2D islets and host non-adapted mites that are exposed to xenobiotic stress. Thus, an evolutionarily conserved response to stress seems to reflect a continuous attempt in both host non-adapted mites and T2D to limit the production of ROS and increase metabolic defense to counter ROS. However, host-adapted mite populations that effectively counteract plant host xenobiotic compounds are able to revert their energy metabolism to normal despite long-term exposure to severe xenobiotic stress. These findings highlight the potential use of energy metabolism as a measure of stress response in mites.

## Results and discussion

### Energy metabolism and stress response pathways are highly conserved between humans and the two-spotted spider mite

To determine the conservation of energy metabolism under xenobiotic stress, the glycolysis, tricarboxylic acid (TCA) cycle, pentose phosphate (PPP), trans-sulfuration (TS), and nicotinamide adenine dinucleotide (NAD) biosynthesis pathways were compared between humans and the two-spotted spider mite (TSSM), *Tetranychus urticae*. The corresponding hsa00010, hsa00030, hsa00020, hsa00270, and hsa00380 KEGG pathways were selected and used to identify a single protein-coding gene for each enzymatic step. In cases where multiple forms of a particular gene existed, the variant encoding the longest human protein sequence was selected. The representative protein sequences were then used as a query against the *Tetranychus urticae* database on ORCAE (https://bioinformatics.psb.ugent.be/orcae/). Mite genes with the bidirectional best hits (BBHs) to human counterparts were considered to be orthologues. The percent similarity, E value, and alignment bit score of mite orthologous genes are shown in Table 1.

**Table 1:** Orthologous genes in energy-generating and stress response pathways between human and *Tetranychus urticae*.

Human protein	Human gene symbol	Human NCBI ID	Mite orthologue exceptions	Mite orthologue gene ID	Bit score	E-value	% identity	% similarity
Glycolysis								
Glucokinase	GCK	2645	–	tetur21g01970	438	5e-148	51	66
Glycogen Synthase	GYS1	2997	–	tetur03g02990	845	0	62	
Glucose-6-phosphate isomerase	GPI	2821	–	tetur02g00660	808	0	69	83
Hypoxia inducible factor 1 subunit-ɑ	HIF1ɑ	3091	–	tetur02g13280	311	4e-90	45	63
6-phosphofructo-2-kinase/fructose-2,6-biphosphatase	PFKFB3	5209	–	tetur11g00350	604	0	64	78
Phosphofructokinase	PFKL	5211	–	tetur12g03690	932	0	60	
Fructose biphosphate aldose	ALDOB	229	–	tetur07g03440	489	2e-173	68	79
Triosephosphate isomerase	TPI1	7167	–	tetur04g06650	347	7e-121	66	78
Glyceraldehyde 3-phosphate dehydrogenase	GAPDH	2597	–	tetur25g00250	434	8e-154	72	81
Phosphoglycerate kinase	PGK1	5230	–	tetur07g07520	558	0	77	85
Phosphoglycerate mutase	PGAM1	5223	Co-factor independent PGAM (iPGAM)	tetur12g03790	N/A	N/A	N/A	N/A
Enolase	ENO1	2023	–	tetur05g08740	671	0	73	86
Pyruvate kinase	PKM	5313	–	tetur13g00130	687	0	65	78
Lactate dehydrogenase	LDHA	3939	No orthologue identified	N/A	N/A	N/A	N/A	N/A
Tricarboxylic acid cycle								
Pyruvate carboxylase	PC	5091	–	tetur05g04260	1626	0	67	81
Pyruvate dehydrogenase kinase 1	PDK1	5163	–	tetur11g03710	118	8e-32	63	76
Pyruvate dehydrogenase E1 subunit alpha 1	PDHA1	5160	–	tetur04g09090	465	4e-163	60	73
Pyruvate dehydrogenase E1 subunit beta	PDHB	5162	–	tetur01g02450	526	0	71	88
Dihydrolipoamide-S-acetyltransferase	DLAT	1737	–	tetur11g02780	482	3e-166	56	71
Dihydrolipoamide dehydrogenase	DLD	1738	–	tetur10g03230	711	0	72	82
Citrate synthase	CS	1431	–	tetur01g06960	662	0	69	83
Aconitase 1	ACO1	48	–	tetur02g09460	1264	0	66	80
Isocitrate dehydrogenase (NAD (+)) 3 catalytic subunit alpha	IDH3A	3419	–	tetur12g00780	509	0	75	85
Isocitrate dehydrogenase (NAD(+)) 3 non-catalytic subunit beta	IDH3B	3420	–	tetur13g01460	488	1e-156	65	79
Isocitrate dehydrogenase (NAD(+)) 3 non-catalytic subunit gamma	IDH3G	3421	–	tetur08g03960	412	4e-142	59	79
Dihydrolipoamide succinyltransferase	DLST	1743	–	tetur08g00510	481	2e-167	64	76
Oxoglutarate dehydrogenase	OGDH	4967	–	tetur01g13490	1295	0	64	77
Succinate-CoA ligase beta subunit	SUCLA2	8803	–	tetur03g02660	372	3e-125	53	68
Succinate-CoA ligase alpha subunit	SUCLG1	8802	–	tetur01g06160	425	4e-149	75	86
Succinate dehydrogenase complex flavoprotein subunit A	SDHA	6389	–	tetur08g03210	998	0	75	85
Succinate dehydrogenase complex	SDHB	6390	–	tetur01g15710	408	1e-143	68	83
Succinate dehydrogenase complex subunit C	SDHC	6391	–	tetur30g00210	84	5e-20	39	52
Succinate dehydrogenase complex subunit D	SDHD	6392	–	tetur20g00790	83	1e-19	43	61
Fumarate hydratase	FH	2271	–	tetur19g01980	658	0	67	79
Malate dehydrogenase cytoplasmic isoform	MDH1	4190	–	tetur12g04290	371	6e-128	58	71
Pentose phosphate pathway								
Glucose 6-phosphate dehydrogenase isoform a	G6PD	2539	–	tetur15g03240	628	0	61	75
6-phosphogluconolactonase	PGLS	25796	–	tetur14g02430	191	1e-59	43	60
6-phosphogluconate dehydrogenase isoform 1	PGD	5226	–	tetur07g04660	757	0	75	87
Ribulose-phosphate 3-epimerase isoform 1	RPE	6120	–	tetur02g08560	302	9e-104	67	83
Ribose 5-phosphate isomerase	RPIA	22934	–	tetur08g00320	90	1e-22	60	73
Transketolase isoform 1	TKT	7086	–	tetur01g09650	780	0	60	76
Transaldolase	TALDO1	6888	–	tetur05g03730	390	5e-133	62	75
Trans-sulfuration pathway								
Cystathionine ß-synthase	CBS	875	–	tetur06g06721	580	0	56	72
Cystathionine ɣ-lyase	CTH	1491	–	tetur23g00710	461	3.00e-161	59	77
Cysteine dioxygenase type 1	CDO1	1036	–	tetur09g00400	203	7.00e-65	46	63
Cysteine sulfinic acid decarboxylase	CSAD	51380	–	tetur19g02930	447	1.00e-150	43	62
Flavin containing dimethylaniline monooxygenase 1	FMO1	2326	–	tetur03g01740	536	1.00e-139	40	59
Cysteine aminotransferase	GOT1	2805	–	tetur08g02390	748	0	55	72
3-mercaptopyruvate sulfurtransferase	MPST	4357	No orthologue identified	N/A	N/A	N/A	N/A	N/A
ɣ-glutamate cysteine ligase	GCLC	2729	–	tetur30g02240	481	1.00e-168	56	73
Glutathione synthetase	GSS	2937	–	tetur34g00580	313	9.00e-101	40	58
NAD+ biosynthesis								
Tryptophan 2,3-dioxygenase	TDO2	6999	–	tetur07g03620	397	3e-136	54	73
Arylformamidase / kynurenine formamidase	AMFID	125061	No orthologue identified	N/A	N/A	N/A	N/A	N/A
Kynurenine 3-monooxygenase	KMO	8564	–	tetur02g03180	445	2.00e-125	48	68
Kynureninase	KYNU	8942	–	tetur16g00150	414	2e-139	48	61
3-hydroxyanthranilate 3,4-dioxygenase	HAAO	23498	–	tetur20g01970	185	3e-56	36	59
Nicotinate-nucleotide pyrophosphorylase	QPRT	23475	–	tetur27g01660	242	3e-78	42	60
Nicotinate phosphoribosyltransferase	NAPRT	93100	–	tetur12g02470	470	2e-160	44	63
Nicotinamide nucleotide adenylyltransferase 1	NMNAT1	64802	Not in genome assembly*	N/A	N/A	N/A	N/A	N/A
NAD synthetase 1	NADSYN1	55191	–	tetur04g01780	497	5e-166	39	57
Nicotinamide phosphoribosyltransferase	NAMPT	10135	Pyrazinamidase/nicotinamidase 1 (PNC-1)	tetur19g00460	N/A	N/A	N/A	N/A
Purine-nucleoside phosphorylase	PNP	4860	–	tetur21g00550	312	1e-105	52	70
Nicotinamide/nicotinate riboside kinase 1	NMRK1	54981	–	tetur03g06790	645	0	64	76

Identification of orthologous genes involved in glycolysis, tricarboxylic acid cycle, pentose phosphate pathway, trans-sulfuration pathway, and nicotinamide adenine dinucleotide biosynthesis pathway was conducted via bidirectional best hit analyses utilizing the Online Resource for Community Annotation of Eukaryotes (ORCAE) database and NCBI BLAST. Bit scores, E values, % identity, and % similarity were recorded for each identifiable orthologue.

*Spider mite homolog of NMNAT1 is not present in the current genome assembly and annotation, but available sequencing data supports its existence.

Despite being distantly related to humans, conservation of the rate limiting glycolysis enzyme—Phosphofructokinase (PFKL)—and other key glycolytic regulators—Glucokinase (GCK), Pyruvate kinase (PK), and Glycogen synthase 1 (GYS) was established in *T. urticae*. Furthermore, the conservation of rate-limiting enzymes Glucose-6 phosphate dehydrogenase (G6PD), Isocitrate dehydrogenase (IDH), and Cystathionine beta-synthase (CBS) was established for the PPP, TCA cycle, and TS pathways, respectively. In addition, the conservation of the rate-limiting enzymes for the Preiss-Handler, *de novo*, and salvage pathways—Nicotinamide nucleotide adenylyltransferase (NMNAT), Tryptophan 2,3-dioxygenase (TDO2) and Quinolinate phosphoribosyltransferase (QPRT), and Nicotinamide phosphoribosyltransferase (NAMPT), respectively - were also confirmed. The high degree of conservation for rate-limiting enzymes between human and *T. urticae* orthologues indicate that these pathways are under considerable evolutionary constraint.

### Comparative analyses reveal species-specific genes involved in energy metabolism and stress response

Despite a high degree of conservation between humans and mite pathways, there are few notable differences in gene complements associated with energy metabolism. Firstly, we were unable to identify the Phosphoglycerate mutase (PGAM) orthologue in mites. In humans, this enzyme is responsible for the conversion of 3-phosphoglycerate (3PG) to 2-phosphoglycerate (2PG) in glycolysis. However, this reaction can be catalyzed by two evolutionarily unrelated and distinct proteins with dissimilar amino acid compositions, catalytic residues, protein structure, and size^29^. The enzyme found in humans is cofactor-dependent PGAM (referred to as PGAM/dPGAM), while the other is cofactor independent PGAM (iPGAM). As the conversion of 3PG to 2PG is an essential reaction in the glycolysis pathway, we looked if mites may be using iPGAM for the catalysis of this reaction. Protein sequences of iPGAM from iPGAM-using organisms (including *Trichoplax adhaerens, Nematostella vectensis, Caenorhabditis elegans, Ixodes scapularis* and *Arabidopsis thaliana*) were retrieved from NCBI and used as a query against the *T. urticae* database to identify orthologues. While an organism may possess a single form or both forms of PGAM, sequence alignments using BLAST confirmed that mites exclusively use the iPGAM form for the conversion of 3PG to 2PG. Secondly, we were unable to identify the orthologue of Lactate dehydrogenase A (LDHA) suggesting that mites cannot ferment pyruvate to form lactate. This confirms the previously reported lack of LDHA activity in mites^30^. These collective findings indicate that the aerobic glycolytic pathway is highly conserved between humans and mites with the exception of generation of lactate from pyruvate. Thirdly, we were unable to identify a mite orthologue of 3-mercaptopyruvate sulfurtransferase (MPST) from the trans-sulfuration pathway. MPST has been lost within several eukaryotic taxa including tunicates and insects^31^. It is not an essential enzyme within the trans-sulfuration pathway as there is a redundancy in the number of enzymes that perform the same function^32^. Fourthly, mites do not have an orthologue of NAMPT that in humans catalyzes the conversion of nicotinamide (NA) to nicotinamide mononucleotide (NAM). Pyrazinamidase/Nicotinamidase 1 (PNC-1) is a functionally equivalent enzyme found in the invertebrate NAD salvage pathway^33^. We identified *tetur19g00460* as an orthologue of the *PNC-1* gene from C*aenorhabditis elegans.* Lastly, Arylformamidase (AFMID), also involved in the NAD biosynthetic pathway, remained undetected in *T. urticae* like in some spiders (*Theridion grallator* and *T. californicum*)^33^.

### The same metabolic endpoints are achieved with different xenobiotic-responsive pathways in humans and the two-spotted spider mite

Metabolic flow through energy-generating pathways is regulated at multiple levels and is modified upon stress. While allosteric inhibition and post-transcriptional modifications regulate important checkpoints in the pathway, here, we compared transcriptional changes within the energy-generating and stress-response pathways between humans and mites when exposed to xenobiotic stress. To reconstruct transcriptional changes in humans, we used expression data from individuals with impaired glucose tolerance (IGT, i.e. prediabetes) and T2D. Transcriptional changes were presented against samples collected from non-diabetic control individuals. On the mite side, we used expression data from host-adapted and host non-adapted mite populations while feeding on one of two challenging hosts, *Arabidopsis thaliana* (thale cress) or *Solanum lycopersicum* (tomato). These data were contrasted against a gene expression set obtained from mites feeding on *Phaseolus vulgaris* (bean), a non-challenging host across mite populations.

#### Aerobic glycolysis

**Figure 2:**
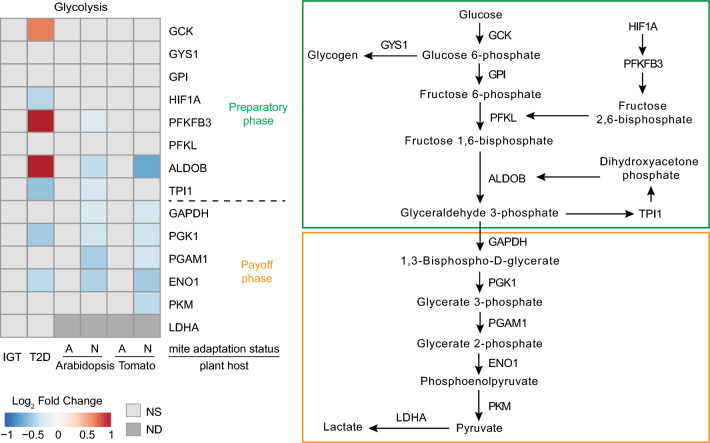
Glycolysis. Gene expression patterns observed in the glycolysis pathway in impaired glucose tolerance (IGT) and type 2 diabetes (T2D) pancreatic β-cells, and adapted (A) and non-adapted (N) spider mites when reared on non-favourable host plants, Arabidopsis and Tomato, relative to a favourable host plant (bean). *NS* not significant at FDR-adjusted p-value cut-off of 0.05, *ND* not detected.

We found no detectable differential stress response between IGT (prediabetic) and non-diabetic samples when we compared the expression of genes encoding enzymes that drive the glycolysis pathway (Fig. 2). Similarly, there were no significant changes in glycolytic gene expression in the Arabidopsis and tomato host-adapted mite samples with the exception of *Triose-phosphate isomerase* (*TPI1)*, downregulated in Arabidopsis-adapted mites (Fig. 2). Lack of differences between IGT and non-diabetics (ND) conditions^28^, highlights the adaptive nature of IGT. By inference, these data indicate that host-adapted mite populations successfully eliminated the effects of host-induced xenobiotic stresses on glucose metabolism. In contrast, T2D and host non-adapted mites show a plethora of transcriptional changes. In human T2D samples there is an upregulation of genes encoding enzymes that drive the preparatory phase of glycolysis. One of them is *Glucokinase* (*GCK*), whose upregulation may affect glucose uptake^34^.In addition, the increased expression of *6-Phosphofructo-2-kinase/Fructose-2,6-biphosphatase 3* (*PFKFB3*) may promote the synthesis of fructose 2,6-bisphosphate (F2,6BP) that is a potent allosteric activator of PFKL, the rate-limiting enzyme of glycolysis^35^. Furthermore, in the T2D sample, there is an upregulation of *Fructose-bisphosphate aldolase* (*ALDOB*) that converts glucose and fructose intermediates to trioses^36^. Based on these expression patterns, one would expect enhanced metabolic flow through the preparatory phase of glycolysis in the T2D sample. This is contrasted by downregulation of genes encoding glycolytic enzymes of the payoff phase such as *Triose-phosphate isomerase*, *Enolase 1,* and *Pyruvate kinase M* (*TPI*, *ENO1,* and *PKM*) indicating suppression of pyruvate metabolism^28^ (Fig. 2). In addition, in diabetic mice and human T2D samples, there is an upregulation of *LDHA*, indicating additional redirection of pyruvate from the mitochondria into lactate generation^19,20^. In xenobiotically-challenged mites, there were no changes in the expression of genes driving the preparatory phase of glycolysis (Fig. 2). However, similar to human T2D, the expression of genes encoding enzymes driving the pay-off phase of the glycolytic pathway was decreased in non-adapted mites upon transfer to either Arabidopsis or tomato (Fig. 2). It appears that mites may have a tighter transcriptional control of pyruvate generation, as genes encoding enzymes of most of the pay-off phase are downregulated. Mites presumably require such a stringency in preventing pyruvate generation under stress as they lack LDHA^30^ that enables a depletion of pyruvate by channeling it toward the synthesis of lactate. Thus, under stress, the generation of pyruvate in both human T2D and non-adapted mite samples seems to be suppressed through downregulation of genes involved in pyruvate generation. A build-up of 6-carbon (C6) glycolytic intermediates in the preparatory phase is expected to be shunted in the PPP^19^ in both humans and mites. Thus, under stress, both human β-cells and mites likely shunt intermediary metabolites by funneling glycolytic precursors such as glucose-6 phosphate (G6P) and fructose-6 phosphate (F6P) towards the PPP for NADPH and GSH production or by build-up of fatty acids, while suppressing pyruvate entry into the mitochondrial TCA cycle.

#### Tricarboxylic acid (TCA) cycle

**Figure 3:**
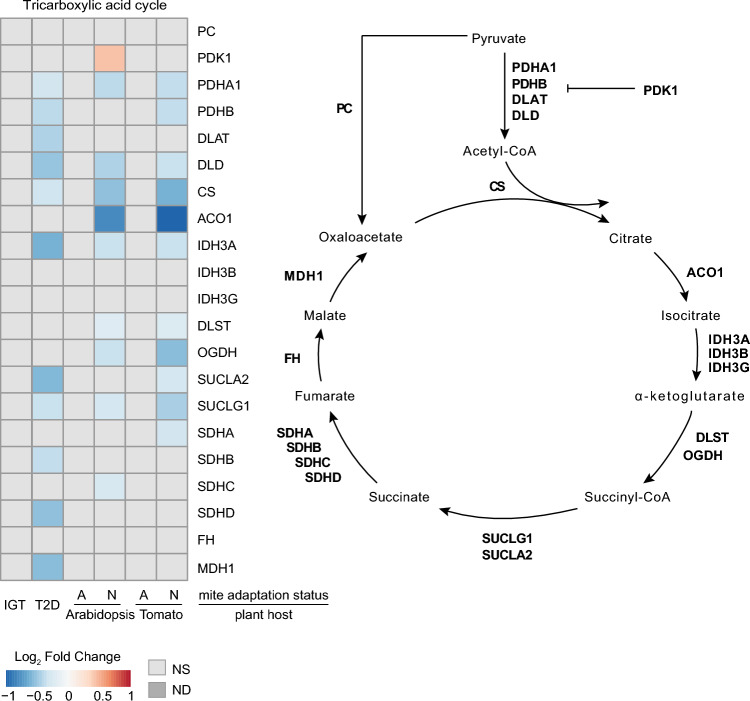
Tricarboxylic acid cycle. Gene expression patterns observed in the tricarboxylic acid cycle pathway in impaired glucose tolerance (IGT) and type 2 diabetes (T2D) pancreatic β-cells, and adapted (A) and non-adapted (N) spider mites when reared on non-favourable host plants, Arabidopsis and Tomato, relative to a favourable host plant (bean). *NS* not significant at FDR-adjusted p-value cut-off of 0.05, *ND* not detected.

Mitochondria can function as a primary source of ROS “leakage” in most tissues and cells^37^. It has been estimated that about 0.2–2% of oxygen consumed is converted into O^2–^ by the electron transport chain^38^ that is interconnected with the TCA cycle. This is particularly augmented under oxidative stress that arises as a consequence of xenobiotic exposure^11^. Therefore, there is a general tendency to reduce utilization of mitochondria in order to limit the impact of oxidative and xenobiotic stresses^20^. Similar to the glycolysis pathway, IGT and host-adapted mite samples did not change the expression of genes encoding enzymes in TCA cycle (Fig. 3). However, T2D and host-non-adapted mite samples showed a similar pattern of gene downregulation that is characteristic of diabetic mouse and human islets where there is transcriptional and protein downregulation of the TCA cycle^19,20^. Remarkably, non-adapted mites on Arabidopsis displayed an elevated *Pyruvate dehydrogenase kinase 1* (*PDK1*) expression similar to diabetic mouse islets^20^ (Fig. 3). Interestingly, *PDK4* in humans similar to *PDK1* in mice and mites, was strongly upregulated in the islets from the donors with T2D^39^. PDK1 and 4 directly block the utilization of pyruvate by the TCA cycle and thus effectively suppress the utilization of this pathway^40^. Decreased expression of genes encoding enzymes of the TCA cycle implied a decline in OxPHOS activity generating ATP, NADH and FADH2, that feed into complexes I, III, and IV of the electron transport chain. The downregulation of the TCA cycle was contrasted by a notable difference in *Oxyglutarate dehydrogenase* (*OGDH*) expression between non-adapted mites and T2D. The OGDH complex catalyzes the decarboxylation of 2-HG to succinyl-CoA and reduces NAD+ to NADH^41^. Under homeostatic regulation, the reducing equivalent NADH feeds the respiratory complex I to generate the mitochondrial membrane potential required for ATP production. Unlike in T2D, in non-adapted mites, OGDH was downregulated, indicating suppression of the mitochondrial metabolism at a larger scale compared to human T2D. Similarly, Drosophila exposed to oxidative stress (that best recapitulates xenobiotic exposure) experienced transcriptional downregulation of pathways enriched for genes responsible for ATP metabolic processes, mitochondrial translation, and generation of precursor metabolites and energy^42^, clearly recapitulating the patterns in mites and T2D^43,44^. The expression pattern of TCA cycle genes suggests that in mites, similar to humans, there is mitochondrial attenuation that slows ATP production in the face of oxidative stress during xenobiotic exposure. The global pattern of mitochondrial attenuation reduces the risk of apoptosis by reducing the risk of ROS accumulation and was shared across the species.

#### Pentose phosphate pathway (PPP)

**Figure 4:**
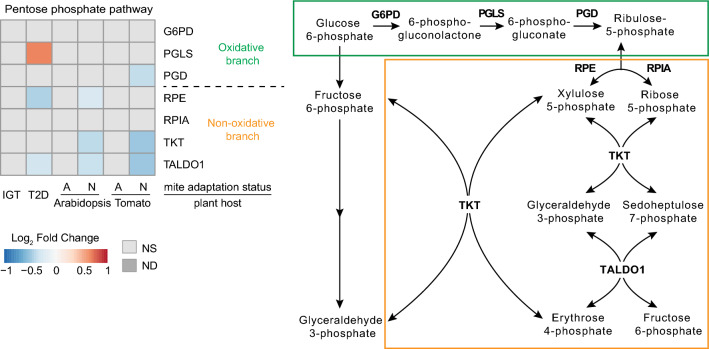
Pentose phosphate pathway. Gene expression patterns observed in the pentose phosphate pathway in impaired glucose tolerance (IGT) and type 2 diabetes (T2D) pancreatic β-cells, and adapted (A) and non-adapted (N) spider mites when reared on non-favourable host plants, Arabidopsis and Tomato, relative to a favourable host plant (bean). *NS* not significant at FDR-adjusted p-value cut-off of 0.05, *ND* not detected.

The attenuation of the pay-off phase of glycolysis and the TCA cycle in T2D and non-host-adapted mite samples is predicted to lead to rewiring of C6 and C3 sugar metabolism. Glucose-6-phosphate (G6P), fructose-6-phosphate (F6P) and glyceraldehyde-3-phosphate (G3P) can be redirected from glycolysis into the oxidative and non-oxidative branches of the pentose phosphate pathway (PPP). In both branches, these metabolites ultimately yield ribose-5-phosphate (R5P)^19,20,45^ (Fig. 4). However, downregulation of *Transketolase* (*TKT*) and *Transaldolase* (*TALDO1*) in T2D and host non-adapted mite samples indicates that the PPP redirects glycolytic flux towards its oxidative branch (Fig. 4). R5P biosynthesis by the oxidative branch also yields NADPH that functions as a crucial antioxidant to quench ROS. NADPH forms reduced glutathione, which converts reactive H_2_O_2_ into H_2_O by glutathione peroxidase^46^. Thus, the synthesis of NADPH is important for the maintenance of cellular redox homeostasis. In human IGT samples and host-adapted mite samples, there were no changes in the expression of genes encoding PPP enzymes (Fig. 4). However, in the T2D sample, increased funneling of glycolytic metabolites into the PPP was supported by increased expression of the gene encoding 6-phosphogluconolactonase (PGLS), the second enzyme in the oxidative branch of the PPP, coinciding with a downregulation of *TALDO1* and *Ribulose-phosphate 3-epimerase* (*RPE*). In non-adapted mites on tomato, the expression of a gene encoding the enzyme Ribose-5 phosphate isomerase A (RPIA) that catalyzes the final step in R5P biosynthesis was increased similar to diabetic mouse islets^20^. This coincides with a downregulation of *TALDO1* and *TKT*, suggesting that mites under stress maximize the synthesis of R5P (Fig. 4). R5P is the main building block for nucleotide synthesis. Given that mites use guanine as their main nitrogen excretion product^47^, coupling ROS detoxification (through the oxidative PPP) with nitrogen excretion may increase the requirement for the PPP in mites under xenobiotic stress. These results suggest that in both human T2D and non-host-adapted mite samples, xenobiotic stress stimulates R5P and NADPH generation that is necessary for nucleotide biosynthesis and antioxidant responses, while restricting the re-entry of PPP intermediates into glycolysis/gluconeogenesis.

#### Trans-sulfuration pathway (TS)

**Figure 5:**
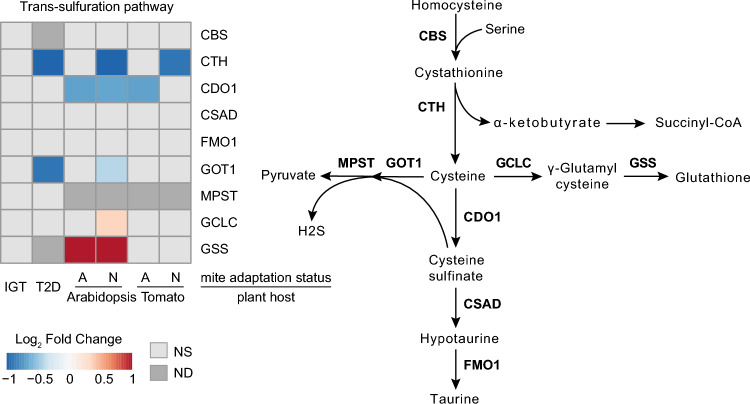
Trans-sulfuration pathway. Gene expression patterns observed in the trans-sulfuration cycle pathway in impaired glucose tolerance (IGT) and type 2 diabetes (T2D) pancreatic β-cells, and adapted (A) and non-adapted (N) spider mites when reared on non-favourable host plants, Arabidopsis and Tomato, relative to a favourable host plant (bean). *NS* not significant at FDR-adjusted p-value cut-off of 0.05, *ND* not detected.

Cysteine availability controls the synthesis of glutathione (GSH), which is the main regulator of cellular redox homeostasis and an important agent in xenobiotic detoxification^48^. The TS pathway does not undergo transcriptional changes in the human IGT sample (Fig. 5). In T2D and host-non-adapted mite samples there is downregulation of the gene encoding Cystathionine gamma-lyase (CTH) that catalyzes the last step in the TS pathway leading to cysteine biosynthesis. Potentially, to preserve the cysteine pool toward the synthesis of GSH, there is coordinated downregulation of genes encoding enzymes that use cysteine toward the synthesis of pyruvate and H_2_S such as Glutamic-oxaloacetic transaminase (GOT1) and Cysteine dioxygenase type 1 (CDO1). A host-specific increase in Glutamate-cysteine ligase catalytic subunit (GCLC) and Glutathione synthase (GSS) expression in Arabidopsis-adapted mites leading to the synthesis of GSH may reflect a need to detoxify Arabidopsis secondary metabolites such as glucosinolates^49^. Even though there are sample/species specific changes in the TS pathway, it appears that cysteine preservation is a priority under diabetogenic and xenobiotic stress.

#### NAD biosynthesis

**Figure 6:**
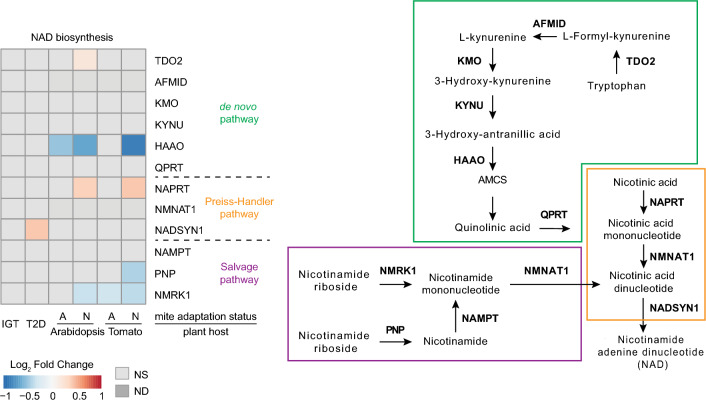
NAD biosynthesis. Gene expression patterns observed in the NAD biosynthesis pathway in impaired glucose tolerance (IGT) and type 2 diabetes (T2D) pancreatic β-cells, and adapted (A) and non-adapted (N) spider mites when reared on non-favourable host plants, Arabidopsis and Tomato, relative to a favourable host plant (bean). *NS* not significant at FDR-adjusted p-value cut-off of 0.05, *ND* not detected.

Reduced or oxidized NAD is a coenzyme for redox reactions in metabolic pathways^50^. It can be synthesized through three different pathways: (a) the Preiss–Handler pathway (PHP), generating NAD from nicotinic acid, (b) the *de novo* synthesis pathway (DNP), generating NAD from tryptophan, and (c) the salvage pathway (SP), generating NAD from nicotinamide (NAM)^8^. In human IGT and host-adapted mite samples, genes encoding enzymes in NAD pathways did not change their expression (Fig. 6). The notable exception is the downregulation of a gene encoding 3-Hydroxyanthranilate 3,4-dioxygenase (HAAO) that is part of the de novo synthesis pathway in Arabidopsis-adapted mites. In non-adapted mites there is a downregulation of genes supporting *de novo* synthesis and salvage pathways (Fig. 6). In addition, the mite orthologue of the human gene encoding Arylformamidase (*Kynurenine formamidase*, AFMID), a key step in tryptophan metabolism, is missing (Table 1). In host non-adapted mites and T2D samples, the synthesis of NAD from nicotinic acid is enhanced through the upregulation of *NAD synthetase 1* (*NADSYN1*) and *Nicotinate phosphoribosyltransferase* (*NAPRT*), respectively, indicating streamlining of NAD biosynthesis from nicotinic acid.

In summary, the comparison of the expression of genes driving cellular energy metabolism between host-adapted and non-adapted mites when transferred to either Arabidopsis or tomato showed that: (a) at the transcriptional level, host non-adapted mites respond to xenobiotic stress similarly to human genes in T2D samples where energy metabolism shifts towards ROS detoxification rather than energy generation, and (b) upon host adaptation, mites revert the expression of genes supporting cellular energy metabolism to a basal level despite the continued presence of xenobiotic compounds in their diet. Therefore, the expression of genes driving cellular energy metabolism can be used as markers of mite host-adaptation status.

### Transcriptional changes in genes involved in energy metabolism early in mite host adaptation process

**Figure 7:**
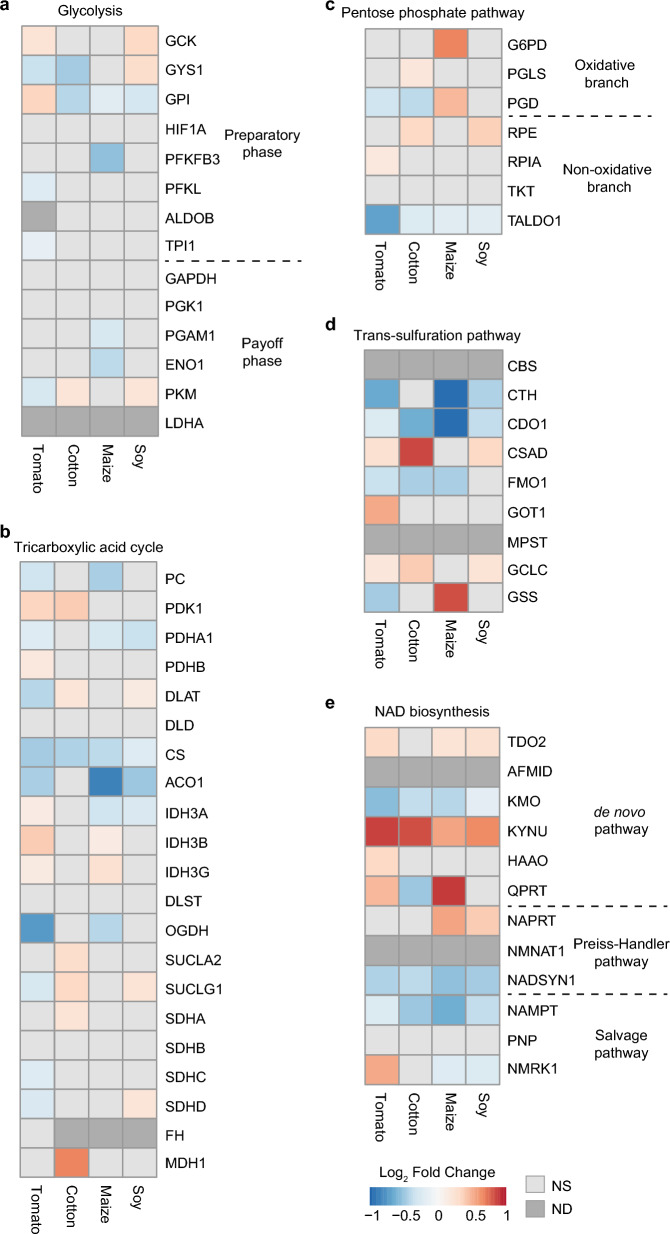
Initial phase of host adaptation in mites. Gene expression patterns observed in energy metabolism pathways of spider mites after short-term adaptation (five generations) to a non-favourable host plant. NS: not significant, ND: not detected. (**a**) Glycolysis. (**b**) Tricarboxylic acid cycle. (**c**) Pentose phosphate pathway. (**d**) Trans-sulfuration pathway. (**e**) NAD biosynthesis. *NS* not significant at FDR-adjusted p-value cut-off of 0.05, *ND* not detected.

Mite adaptation to a new plant host occurs over 5–25 generations^23,25,26^. Samples used in our analysis so far were collected from host-adapted mites that were maintained on “new” plant hosts for >25 generations. To determine trends in energy metabolism early in the adaptation process we used gene expression data collected from mites that were exposed to a new host for ~5 generations. At that time, there is already a significant improvement in mite fitness relative to non-adapted mites^25,26^ and the general gene expression pattern shows similarity to mite populations that are resistant to xenobiotic stress^51^. Figure 7 shows gene expression across five energy and stress metabolism pathways in mites that were maintained on tomato, cotton, maize and soybean leaves for ~5 generations (5G). The expression of selected genes in 5G mites differs from patterns seen in the host-adapted mite samples and have greater similarity to responses observed in host non-adapted mites and the T2D sample. Specifically, upregulation/basal levels of GCK (the first step in the glycolysis pathway) suggests the commitment of glucose for retention in cells and entry into the glycolysis pathway. Even though the expression of genes driving the glycolysis pathway are not downregulated to the same level as seen in T2D and host non-adapted mite samples, the upregulation of genes in the PPP that sequester glucose-6-phosphate away from glycolysis suggests that like in human T2D and mite xenobiotically stressed samples, 5G mites still favour detoxification of ROS and the production of R5P at the cost of production of pyruvate. This is consistent with the downregulation of genes that enable pyruvate entry from glycolysis into the TCA cycle (Fig. 7A–C). Consistent with the increased metabolite flow through the PPP and similarly to T2D and mite host non-adapted samples, the expression of genes supporting the trans-sulfuration pathway suggests channeling of cysteine toward the generation of glutathione (Fig. 7D). The expression of genes from NAD biosynthetic pathways suggests that the Preiss-Handler pathway remains equally important for 5G mites as it is for humans under xenobiotic stress (Fig. 7E).

While there is a good parallelism in gene expression patterns across the five pathways between T2D, 5G, and mite host non-adapted samples, 5G mite samples show additional interesting gene expression profiles within the TCA and NAD pathways. In the TCA cycle, 5G mites have an increased expression of genes encoding either Dihydrolipoamide acetyltransferase (DLAT), a subunit of the multienzyme complex Pyruvate dehydrogenase (PDH)^52^, Isocitrate dehydrogenases (IDH3), Succinate dehydrogenase (SDH, iron sulfur subunits), or mitochondrial Malate dehydrogenase (MDH1) (Fig. 7B). In humans, these enzymes have broader functions beyond the TCA cycle. For example, MDH2 and IDH1 are promiscuous and able to catalyze the alpha-ketoglutarate (α-KG) conversion to L-2-hydroxyglutarate (L-2-HG)^53^ that in turn inhibit the activity of Ten-Eleven Translocation (TET) Methylcytosine dioxygenases, enzymes associated with DNA demethylation^54^. In addition to this route of L-2-HG synthesis, in host non-adapted mites and some 5G samples, there is a downregulation of OGDH that also leads to the accumulation of L-2-HG, indicating potential enhancement of L-2-HG synthesis in 5G mites. Besides, upregulation of either *IDH3* or *SUCL* in 5G mites may reflect their enhanced capacity to deal with ROS, as IDH1 in humans is involved in maintenance of reduced glutathione^55^ while SUCLG1 and SUCLA2 are implicated in protein expression of redox-scavenging enzymes^56^. Furthermore, within the *de novo* NAD synthesis sub pathway, there is an upregulation of a heme-dependent dioxygenase (*TDO2*) that catalyzes the oxidative cleavage of L-tryptophan (L-Trp) and downregulation of downstream enzyme Kynurenine 3-monooxygenase (KMO) (Fig. 7E). This expression pattern is predicted to yield greater amounts of kynurenine in 5G mites. In humans, kynurenine activates Aryl hydrocarbon receptor (AhR) and causes oxidative stress and pro-inflammatory responses^57^. Interestingly, Kynureninase (KYNU), an enzyme that catalyzes the cleavage of L-kynurenine and is involved in biosynthesis of NAD, is strongly upregulated in all 5G mites, indicating that kynurenine pathway in 5G mites is diverted into *de-novo* NAD synthesis away from AhR signaling. Collectively, diversion of kynurenine pathway into NAD biosynthesis and the upregulation of selected TCA enzymes may indicate enhancement of antioxidant defenses and epigenetic changes in gene regulation in 5G mites.

## Conclusion

We used gene expression data to compare transcriptional responses of five pathways—glycolysis, pentose phosphate (PPP), tricarboxylic acid (TCA) cycle, trans-sulfuration (TS), and NAD biosynthesis—between host-adapted and host non-adapted spider mite populations using responses in pancreatic β-cells of type-2 diabetes patients to model energy metabolism under stress. All five pathways were highly conserved between spider mites and humans, with the exception of some species-specific differences in lactate production, the TS pathway, and NAD biosynthesis. Gene expression data revealed similar responses to diabetogenic stress and host-plant transfer between humans and spider mites, respectively. The payoff phase of glycolysis and the TCA cycle were downregulated in both samples, likely to prevent excess reactive oxygen species (ROS) production during aerobic respiration. Similarly, genes required to shunt carbon back to glycolysis are downregulated in the PPP, while genes supporting antioxidant production in the PPP and TS pathway were upregulated. Gene expression data would also suggest a preference for NAD biosynthesis from nicotinic acid in both T2D and non-adapted mite samples. Remarkably, our data show that in both host-adapted mite strains, gene expression returns to a basal level in each pathway. Only one notable host-specific difference was observed, where the glutathione synthetase gene of the TS pathway is upregulated only in Arabidopsis-adapted mites, likely reflective of their requirement to detoxify Brassicaceous defense compounds, glucosinolates. Therefore, upon host adaptation, mites revert the expression of genes supporting energy metabolism to normal despite the continued presence of xenobiotics in their diet. We also examined mite gene expression changes after short-term adaptation to a new host. After five generations on a new host, mites still experienced dysregulation at the transcriptional level in each pathway to favour ROS detoxification over energy generation, which parallels the responses seen in T2D and host non-adapted samples. These findings were in line with a previous study that demonstrated a notable decrease in the transcription of genes linked to gluconeogenesis and the generation of ATP in mites subjected to spatiotemporal stress and selection^58^. Collectively, our data show that upon host-adaptation, mites can overcome the metabolic stress associated with host-plant transfer, suggesting that genes involved in energy metabolism can serve as molecular markers for mite host-adaptation.

## Materials and methods

### Identification of human metabolic pathways and protein sequences

The *Homo sapiens* KEGG database was used to identify all protein-coding genes involved in metabolism associated with glucose homeostasis. Enzymes in the glycolysis, pentose phosphate, citric acid, trans-sulfuration and NAD pathways were retrieved using KEGG IDs hsa00010, hsa00030, hsa00020, hsa00270 and hsa00380, respectively. A representative protein sequence for each enzyme was retrieved from the NCBI database.

### BLAST based identification of mite orthologues

Human sequences were used as a query against the *Tetranychus urticae* protein database using the blastp program^59^ with BLOSUM62 similarity matrix. The top scoring matches with bit score of at least 50, E-value below 1E-4, and percent similarity higher than 50 were used in reciprocal blastp searches against human protein database to establish bidirectional best hits (BBH) orthology^60^.

### Mites and plant host maintenance and sample preparation

Tomato, *Solanum lycopersicum* cv Moneymaker, and bean, *Phaseolus vulgaris* cv California Red Kidney, plants were grown in growth chambers at 25 °C, 60 % relative humidity and with a 16:8 h light:dark photoperiod. Reference mite population *Tetranychus urticae* “London” was reared on bean plants for >10 years. The *T. urticae* tomato and Arabidopsis adapted strains were derived from “London” via experimental selection and maintained on tomato cv Moneymaker and Arabidopsis Col-0 respectively^23,61^. For all experiments, adult female mites were used. Both non-adapted and adapted mites were reared in the common garden (bean) for two generations prior to host transfer experiment. Subterminal leaflets of 4–5 weeks old tomato plants were infested with 100 adult female mites. After 24 hours, mites were collected and samples from two plants were pooled to produce a single biological replicate.

Experimental plants used in the study were either commercial varieties (tomato, soy, maize, cotton) or laboratory strain (Arabidopsis, seed obtained from The Arabidopsis Information Resource). None of them are endangered species. We confirm that all methods were carried out in accordance with relevant guidelines in the method section.

### RNA extraction and sequencing following tomato host plant transfer

RNA extraction was performed using the RNeasy Mini Kit followed by an on-column DNase treatment to ensure genomic DNA removal. The quantity and purity of extracted RNA was assessed using a Thermo Scientific NanoDrop 2000. Strand specific paired-end (2 × 75 bp) sequencing was conducted according to Illumina TruSeq protocol (Illumina, San Diego, CA) at the Centro Nacional de Análisis Genómico (CNAG) (Barcelona, Spain). Reads were mapped to the reference *T. urticae* genome^27^ using STAR aligner v.2.7.1a^62^ in a two-pass mode with annotation allowing only unique mapping, up to five mismatches per read mapped, a minimum intron size of 20 bp, a maximum intron size of 15,000 bp. Read counts were generated at the level of gene locus using HTSeq v.0.6.0 in “union” mode^63^ against *T. urticae* genome yielding 9–13.5 million mapped fragments per library. Genes expressed at the level at or above 1 fragment count per million reads in at least three samples were considered for the subsequent analysis. Log2 fold changes and associated FDR-adjusted p-values were generated using voom/limma workflow^64^.

### Expression data retrieval and processing

RNA-Seq expression data for human control, impaired glucose tolerance (IGT), and type 2 diabetes (T2D) pancreatic islets were retrieved from the GEO accession GSE164416. Data pre-processing and normalization was conducted as per original publication^28^. Briefly, read counts were retrieved from the GEO public database, genes were filtered by mean raw read count over 5, and samples with INS (ENSG00000254647) as the highest expressed gene were retained for subsequent analysis using voom/limma workflow^64^ to generate Log2 fold changes and associated adjusted p-values.

RNA-Seq data for adapted and non-adapted spider mites transfer to Arabidopsis were retrieved from NCBI SRA BioProject PRJNA701185. Data pre-processing and normalization was conducted as per original publication^65^ and as described above for the tomato-associated RNA-Seq dataset. Log2 fold changes and FDR-adjusted p-values for RNA-Seq expression data were generated using voom/limma workflow^64^.

Microarray gene expression data for spider mites transfer acclimated to soybean, maize, and cotton was retrieved from GEO accession GSE80337^66^, and tomato from GEO accession GSE39869^51^. Raw intensity data was background corrected by the ‘normexp’ method, with an offset of 50. Background-corrected data were within- and between-array normalized (global loess and Aquantile, respectively)^67^. Prior to gene expression analysis, the probe sequences were remapped to the *T. urticae* cDNA sequences of January 25, 2019 retrieved from ORCAE^68^ using Bowtie 2 version 2.4.5 with default settings^69^. Log2 fold changes and FDR-adjusted p-values were generated using limma workflow^70^.

### Comparison of gene expression changes across data sets

Log2 fold changes for metabolic pathways genes from human and mite RNA-Seq and microarray data were filtered at FDR-adjusted p-value cut-off of 0.05 and visualized using pheatmap R package^71^.

## Data Availability

Sequencing data used in this study were deposited to NCBI SRA under BioProject PRJNA1020863.
